# Application of 4D-QSAR Studies to a Series of Raloxifene Analogs and Design of Potential Selective Estrogen Receptor Modulators

**DOI:** 10.3390/molecules17067415

**Published:** 2012-06-15

**Authors:** Ana Carolina Rennó Sodero, Nelilma Correia Romeiro, Elaine Fontes Ferreira da Cunha, Uiaran de Oliveira Magalhães, Ricardo Bicca de Alencastro, Carlos Rangel Rodrigues, Lúcio Mendes Cabral, Helena Carla Castro, Magaly Girão Albuquerque

**Affiliations:** 1 Laboratory of Molecular Modeling (LabMMol), Program of Post-Graduation in Chemistry (PPGQu), Institute of Chemistry, Federal University of Rio de Janeiro (Universidade Federal do Rio de Janeiro, UFRJ), Rio de Janeiro 21949-900, RJ, Brazil; 2 Laboratory of Molecular Modeling & QSAR-3D (ModMolQSAR), Faculty of Pharmacy, UFRJ, Rio de Janeiro 21941-599, RJ, Brazil; 3 UFRJ, Campus UFRJ-Macaé, Macaé 27901-000, RJ, Brazil; 4 Department of Chemistry, Federal University of Lavras (Universidade Federal de Lavras, UFLA), University Campus, Lavras 37200-000, MG, Brazil; 5 Laboratory of Industrial Pharmaceutical Technology (LabTIF), Faculty of Pharmacy, UFRJ, Rio de Janeiro 21941-590, RJ, Brazil; 6 Laboratory of Antibiotics, Biochemistry, Education and Molecular Modeling (LABiEMol), Institute of Biology (IB), Fluminense Federal University (Universidade Federal Fluminense, UFF), Campus of Valonguinho, Niterói 24210-130, RJ, Brazil

**Keywords:** four dimensional quantitative structure-activity relationship (4D-QSAR), ligand based drug design (LBDD), molecular modeling, estrogen receptor alpha (ERα), estrogen receptor beta (ERβ), selective estrogen receptor modulator (SERM), raloxifene, ligand binding domain (LBD)

## Abstract

Four-dimensional quantitative structure-activity relationship (4D-QSAR) analysis was applied on a series of 54 2-arylbenzothiophene derivatives, synthesized by Grese and coworkers, based on raloxifene (an estrogen receptor-alpha antagonist), and evaluated as ERα ligands and as inhibitors of estrogen-stimulated proliferation of MCF-7 breast cancer cells. The conformations of each analogue, sampled from a molecular dynamics simulation, were placed in a grid cell lattice according to three trial alignments, considering two grid cell sizes (1.0 and 2.0 Å). The QSAR equations, generated by a combined scheme of genetic algorithms (GA) and partial least squares (PLS) regression, were evaluated by “leave-one-out” cross-validation, using a training set of 41 compounds. External validation was performed using a test set of 13 compounds. The obtained 4D-QSAR models are in agreement with the proposed mechanism of action for raloxifene. This study allowed a quantitative prediction of compounds’ potency and supported the design of new raloxifene analogs.

## 1. Introduction

**Figure 1:**
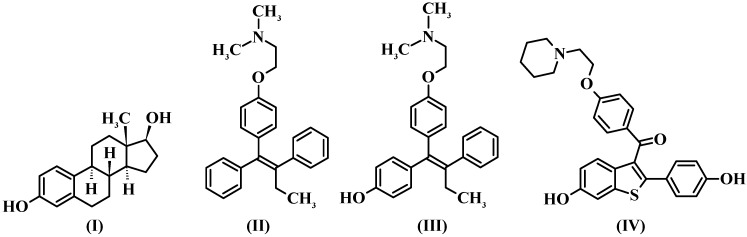
Structures of 17β-estradiol (**I**), tamoxifen (**II**), 4-hydroxy-tamoxifen (**III**) (an active metabolite of tamoxifen), and raloxifene (**IV**).

Estrogens (e.g., 17β-estradiol, **I**, Figure 1) are steroid hormones, synthesized from cholesterol [1], that play a major role in female reproductive function and in cardiovascular and central nervous systems [2]. Estrogenic responses are mediated by specific nuclear receptors named Estrogen Receptors (ERs), which exist in two subtypes: Estrogen Receptor alpha (ERα) and Estrogen Receptor beta (ERβ). Hormone binding to ERs induces the activation or repression of genes [3].

Estrogen replacement therapy, in postmenopausal women, prevents osteoporosis and has advantageous effects on the cardiovascular system [4,5,6]. However, this therapy can increase the risk of hormone-dependent breast and uterine cancer [7]. In this regard, alternative treatments have been studied and new drugs have been developed, such as those agents classified as Selective Estrogen Receptor Modulators (SERMs), because they act, according to the target tissue, as estrogen agonists and antagonists, e.g., inhibiting bone resorption and breast cancer growth [1,2,8]. Details about SERMs’ mechanism of action are described by Dutertre and Smith [8]. Tamoxifen (**II**, Figure 1), the first-generation SERM approved by the U.S. Food and Drug Administration (FDA) agency and the most widely used drug for breast cancer treatment and prevention, displays estrogen agonist effects in bone tissue and cardiovascular system, and acts as an estrogen antagonist in breast but, on the other hand, it manifests partial estrogen agonist activity in the uterus [1,9,10]. Tamoxifen is mainly metabolized to 4-hydroxytamoxifen (**III**, Figure 1), an active metabolite, which has greater ER affinity than the parent drug [4]. Raloxifene (**IV**, Figure 1), a second-generation SERM approved by the FDA agency for prevention of osteoporosis in postmenopausal women, is distinguished from tamoxifen by its lack of proliferative effects in uterine tissue [11,12] and is being evaluated to treat and prevent breast cancer, osteoporosis, and cardiovascular diseases [13,14].

Grese and co-workers [11] described four important structural features of raloxifene when compared to tamoxifen and 4-hydroxytamoxifen (Figure 1): (i) the two phenolic hydroxyl groups; (ii) different substituents on the basic aliphatic amine group; (iii) incorporation of the stilbene moiety into the 2-phenyl-1-benzothiophene framework; and (iv) the carbonyl “hinge” between the side chain containing the basic aliphatic amine and the benzothiophene aromatic ring. These structural elements give raloxifene a distinct molecular conformation, which may affect the conformation of the raloxifene-ER complex, probably being responsible for the unique tissue selectivity observed for this compound.

**Figure 2:**
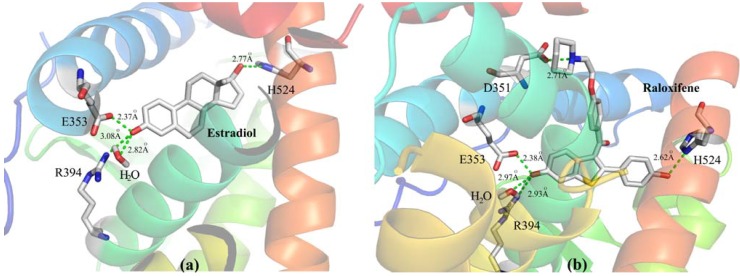
Schematic representation of the X-ray complex formed by the LBD of ERα with: (**a**) estradiol (PDB code: 1ERE) and (**b**) raloxifene (PDB code: 1ERR).

The crystal structures of ERα in complex with 17β-estradiol (the endogenous estrogen) and raloxifene [3] are available in the Protein Data Bank (PDB) [15]. These crystallographic structures show that raloxifene (PDB code: 1ERR) binds at the ligand binding domain (LBD), *i.e.*, the same binding site of estradiol (PDB code: 1ERE) (Figure 2) [3]. Raloxifene is anchored to the LBD of ERα by a direct hydrogen-bonding network comprising (Figure 2): the nitrogen atom (feature II, Figure 1) of the piperidine ring with Asp351; the phenolic hydroxyl group (feature Ia) with Glu353 and Arg394; and the phenolic hydroxyl group (feature Ib) with the imidazole nitrogen atom of His524 [3].

The side chain of raloxifene makes extensive hydrophobic interactions, but it is too long (over 11 Å) to be contained within the confines of the binding cavity. Therefore, the piperidine moiety displaces helix H12 and protrudes out of the pocket between helices H3 and H11 (Figure 2). Brzozowski and co-workers [3] argue that the antagonistic response of raloxifene is based on its ability to prevent the formation of an active conformation of the raloxifene-ERα complex, which is dependent on H12.

Based on the structure of raloxifene, Grese and coworkers [16] synthesized and evaluated a series of 2-arylbenzothiophene derivatives as ligands of the ERα and as inhibitors of the MCF-7 breast cancer cell proliferation *in vitro*. In the current work, we developed four-dimensional quantitative structure-activity relationship (4D-QSAR) [17,18,19,20,21] models for this series of 2-arylbenzothiophene derivatives, in order to better understand the mechanism of action of this class of compounds and to design new raloxifene analogs as potential selective estrogen receptor modulators.

## 2. Results and Discussion

### 2.1. Grid Cell Size and Alignment Evaluation

**Figure 3:**
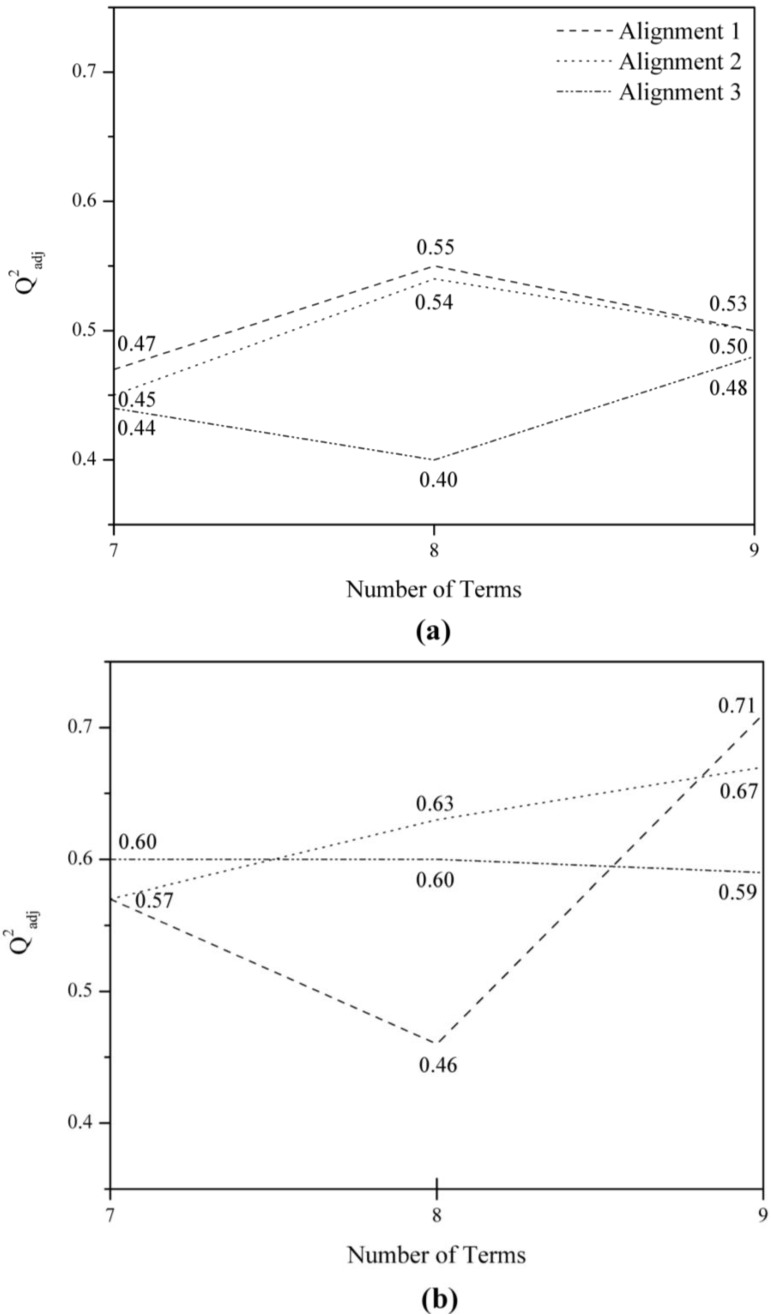
Plots of Q^2^_adj_ values *versus* number of descriptors (terms) in the best models for Alignment 1 (---), Alignment 2 (∙∙∙) and Alignment 3 (-∙∙-), using grid cell sizes of (**a**) 2.0 and (**b**) 1.0 Å.

In order to identify the best grid cell size and alignment, we plotted the adjusted cross-validated R^2^ (Q^2^_adj_) values *versus* the number of terms included in the corresponding equation, according to the two grid cell sizes (2.0 and 1.0 Å) and the three alignments considered (Figure 3). Besides, to define the number of descriptors that should be included in a good predictive model, we analyzed models with seven, eight, and no more than nine terms, avoiding possible data overfitting [22].

The best models generated by 1.0 Å grid cell are more predictive (higher Q^2^_adj_ values) than the best models from 2.0 Å grid cell (Figure 3), irrespective to the alignment. Although alignment 3 had shown good performance, a preliminary analysis of those models demonstrated that the spatial localization of their selected descriptors (GCODs) (data not shown) is not consistent with the ER modulators action mechanism. Therefore, only alignments 1 and 2, obtained with a grid cell size of 1.0 Å, will be discussed from this point forward.

### 2.2. Best Models from Alignment 1

The best models **1B7** and **1B9** (1.0 Å grid cell) are described in Table 1. Model **1B8** was eliminated from the analysis because it presented a low Q^2^_adj_ value (<0.5) (Figure 3). In order to determine if the information in models **1B7** and **1B9** is redundant, the correlation coefficient (R) of their residuals was calculated (*i.e.*, pIC_50_ experimental—pIC_50_ calculated). Equivalent models typically have nearly the same distribution of residuals (*i.e.*, R ≈ 1) and independent models will have nearly uncorrelated residuals (*i.e.*, R ≈ 0) [23]. The results show that models **1B7** and **1B9** have some degree of correlation (R = 0.59), probably due to the presence of spatially identical grid cells with the same IPE type (Table 1), namely GCODs (**2,11,4**)(**any**), **(−1,8,2**)(**any**), and (**1,13,1**)(**any**).

**Table 1:** Best models of Alignment 1 obtained from 1.0 Å grid cells.

Model	Terms	Equation ^a^
**1B7**	7	pIC_50_ = 7.89 − 13.99 (**2,11,4**)(**any**)− 6.96 (**−1,8,2**)(**any**) − 2.04 (**0,−2,9**)(**any**)
		+ 22.68 (**1,13,1**)(**any**)+ 12.16 (**1,−1,9**)(**any**)
		+ 1.84 (**1,2,2**)(**ar**) + 19.60 (**1,11,−2**)(**any**)
**1B9**	9	pIC_50_ = 8.19 − 18.84 (**2,11,4**)(**any**) − 6.13 (**0,−2,−1**)(**any**) + 8.45 (**0,1,−2**)(**any**)
		− 8.71 (**0,11,5**)(**any**) − 7.54 (**−1,8,2**)(**any**) − 20.98 (**1,6,−2**)(**np**)
		+ 15.96 (**1,13,1**)(**any**) + 3.93 (**1,2,0**)(**any**) + 1.61 (**0,0,−2**)(**any**)

^a^ The three numbers inside the first parenthesis defines the GCOD’s Cartesian coordinates (x,y,z), and the letters inside the second parenthesis defines the atom type occupancy (IPE).

We also computed the cross-correlation matrix of the GCODs from models **1B7** and **1B9** (Table 2) to determine if two or more highly correlated GCODs appear in the same 4D-QSAR model. According to Table 2, except only for two pairs of cells, the other pairs of descriptors are poorly correlated (R < 0.5). This means that each of these descriptors contributes in different ways to the 4D-QSAR models [22]. The highest correlations occur between the pair of GCODs (**−1,8,2**)(**any**) and (**1,13,1**)(**any**) (R = 0.52) and also between the pair of GCODs (**0,−2,−1**)(**any**) and (**0,1,−2**)(**any**) (R = 0.47). The first pair of GCODs may be found in both models (**1B7** and **1B9**), while the second pair is found only in model **1B9**.

**Table 2:** Cross-correlation matrix of the GCODs and the experimental pIC_50_ values of models **1B7** and **1B9**.

	Potency	(2,11,4) (any)	(0,−2,−1) (any)	(0,1,−2) (any)	(0,11,5) (any)	(−1,8,2) (any)	(1,6,−2) (np)
**Potency**	1.00						
**(2,11,4)** **(any)**	−0.46	1.00					
**(0,−2,−1)** **(any)**	−0.31	0.13	1.00				
**(0,1,−2)** **(any)**	−0.26	0.36	0.47	1.00			
**(0,11,5)** **(any)**	−0.35	0.30	0.01	0.13	1.00		
**(−1,8,2)** **(any)**	−0.27	0.20	0.10	0.12	−0.17	1.00	
**(1,6,−2)** **(np)**	−0.21	−0.21	−0.07	−0.13	−0.21	0.06	1.00
**(1,13,1)** **(any)**	0.12	0.13	0.00	−0.07	−0.37	0.52	0.01
**(1,2,0)** **(any)**	0.36	−0.01	0.18	0.18	−0.16	0.22	0.11
**(0,0,−2)** **(any)**	0.43	−0.31	0.26	−0.19	0.01	−0.11	0.07
**(0,−2,9)** **(any)**	−0.30	0.05	0.20	0.16	−0.29	0.18	−0.05
**(1,−1,9)** **(any)**	0.36	−0.26	−0.11	−0.08	−0.11	−0.09	0.06
**(1,2,2)** **(ar)**	0.33	−0.18	-0.27	0.21	0.14	−0.16	−0.09
**(1,11,−2)** **(any)**	0.60	−0.24	−0.10	−0.17	−0.41	−0.06	−0.27
	**(1,13,1)** **(any)**	**(1,2,0)** **(any)**	**(0,0,−2)** **(any)**	**(0,−2,9)** **(any)**	**(1,−1,9)** **(any)**	**(1,2,2)** **(ar)**	**(1,11,−2)** **(any)**
**(1,13,1)** **(any)**	1.00						
**(1,2,0)** **(any)**	0.10	1.00					
**(0,0,−2)** **(any)**	−0.13	0.28	1.00				
**(0,−2,9)** **(any)**	0.12	−0.11	−0.35	1.00			
**(1,−1,9)** **(any)**	−0.27	0.41	0.35	−0.23	1.00		
**(1,2,2)** **(ar)**	−0.17	0.34	0.06	−0.18	0.35	1.00	
**(1,11,−2)** **(any)**	0.06	0.08	0.31	0.07	0.02	−0.08	1.00

The cross-correlation matrix of the experimental pIC_50_ values and the frequency of grid cell occupancy of models **1B7** and **1B9** were calculated (Table 2). It has been demonstrated that the highest individual correlation with activity, except only for GCOD (**1,11,−2**)(**any**), is shown by GCODs (**2,11,4**)(**any**) (R = −0.46), (**0,0,2**)(**any**) (R = 0.43), (**1,2,0**)(**any**) (R = 0.36), and (**0,11,5**)(**any**) (R = −0.35), which are present only in model **1B9**.

Outliers were defined as those compounds whose residuals are higher than twice the standard deviation of the residual of fit (SD_res_). The standard deviations were computed for the residuals of all 41 compounds of the training set for models **1B7** (SD_res_ = 0.53) and **1B9** (SD_res_ = 0.38). The results show that model **1B9** presents lower SD_res_ value and, consequently, only compound **9** was defined as an outlier. On the other hand, model **1B7** shows a higher SD_res_ value and presents three outliers (namely, compounds **2**, **6**, and **13**).

### 2.3. Graphical Analysis of the Representative Model of Alignment 1

**Figure 4:**
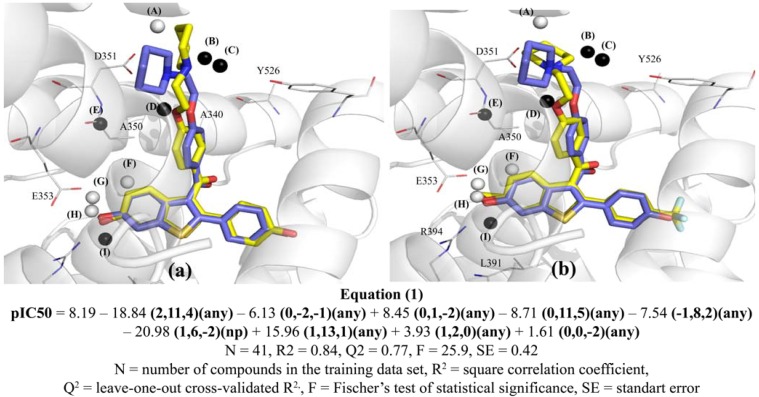
Graphical representation of compounds **1** (**a**) and **54** (**b**) according to the 4D-QSAR Model **1B9** (Alignment 1, grid cell size of 1.0 Å, and 9 terms). The postulated “bioactive” conformations of compounds **1** and **54** (stick models in yellow) were superposed (according Alignment 1) to the X-ray structure of raloxifene (stick models in blue) bound in the LBD of ERα (secondary structure and line models of selected amino acid residues in gray). GCODs of Model **1B9** are: (A) = (**1,13,1**)(**any**); (B) = (**2,11,4**)(**any**); (C) = (**0,11,5**)(**any**); (D) = (**−1,8,2**)(**any**); (E) = (**1,6,−2**)(**np**); (F) = (**1,2,0**)(**any**); (G) = (**0,1,−2**)(**any**); (H) = (**0,0,−2**)(**any**); and (I) = (**0,−2,−1**)(**any**). The white and black spheres represent the GCODs which occupation contribute to increase (GCODs A, F–H) or to decrease (GCODs B–E, and I) the potency of the compounds.

Since Model **1B9**, as described in Equation 1 (Figure 4), exhibits higher R^2^_adj_ and Q^2^_adj_ values, and fewer number of outliers than Model **1B7**, it was selected as the representative model. According to the cross-correlation matrix of the Model **1B9** GCODs (Table 2), grid cells (**1,13,1**)(**any**) and (**−1,8,2**)(**any**) seem to partially supply (R = 0.52) the same type of structure-activity information, in spite of the fact that these cells are spatially distant (5.48 Å) and have opposite contributions. The GCOD (**1,13,1**)(**any**) occupation increases the compounds potency, while the GCOD (**−1,8,2**)(**any**) occupation decreases. Both descriptors are located close to the side chain of the arylbenzothiophenes, being related to the flexibility of this basic side chain. In fact, GCOD (**1,13,1**)(**any**) is close to the carbon atom (C4) of the piperidine ring, while GCOD (**−1,8,2**)(**any**) is close to the oxygen atom of the ethoxy-phenyl group (Figure 3).

This correlation is probably due to an intramolecular hydrogen bonding (N∙∙∙O distance = 3.02 Å), reinforced by a strong attractive electrostatic interaction between the corresponding amino and ethoxyl groups. This interaction causes a preferential synclinal conformation of this lateral chain, involving the four atoms of the N-C-C-O moiety. Therefore, since GCOD (**1,13,1**)(**any**) is the descriptor that most contributes to increase the potency (coefficient value = 15.96, Equation 1, Figure 4), this fact indicates the relevance of the synclinal conformation of the basic side chain for the structure-activity relationship (SAR) of this series of compounds.

The cells (**2,11,4**)(**any**) and (**0,11,5**)(**any**) are close to each other and present negative coefficients, being also related to the basic side chain, located near the carbon (C2) and nitrogen atoms of the piperidine ring, respectively (Figure 4). Altogether, those four GCODs indicate a preferential orientation of the piperidine side chain. This group is involved in an intermolecular hydrogen bond, intensified by an electrostatic interaction with Asp351, which is corroborated by Wang and co-workers [24]. The authors employed a series of compounds, structurally related to raloxifene, in construction of a 2D-QSAR model and proposed that hydrogen bonds are important, but not an unique feature to binding affinity. That orientation is essential to increase or decrease the potency of the raloxifene analogs. Additionally, the basic side chain of raloxifene also makes extensive hydrophobic contacts with the alpha helixes H3, H5/6, H11 and the loop between the alpha helixes H11 and H12 [3], reinforcing the importance of the orientation and conformation of this basic side chain.

The GCODs (**0,1,−2**)(**any**) and (**0,−2,−1**)(**any**) can also be considered to contain some degree of similarity (R = 0.47). In spite of presenting opposite contributions, these cells are located close to each other (3.16Å), partially leading to the same type of information. The GCODs (**1,2,0**)(**any**), (**0,1,−2**)(**any**), (**0,0,−2**)(**any**), and (**0,−2,−1**)(**any**) reflect the importance of the hydrogen-bonding network close to the benzothiophenyl moiety for the antagonist activity of the ERα ligands. In fact, the cell (**0,0,−2**)(**any**) is directly related to the hydrogen bonding interactions of the 6-OH group of the benzothiophene ring with Glu353 and Arg394, as described in previous SAR studies [3,11,16,25]. The occupation of this cell is drastically reduced when this position is non-substituted, or the substituents are unable to perform those hydrogen bond interactions (e.g., **23**, **30**, **31**, **35**, **38**, and **41**). The same happens with GCOD (**0,1,−2**)(**any**), since compounds with hydrogen bond acceptor substituents at C6-position have a high frequency of occupation (e.g., **37**, **49**, and **50**).

The X-ray crystal structure of the raloxifene-ERα complex shows the benzothiophene ring of raloxifene surrounded by hydrophobic residues, such as Leu349, Ala350, Leu387, Leu391 and Phe404 [3]. Therefore, the occupation by any atom types in grid cell **(0,−2,−1)** decreases the potency of the compounds due to steric factors. This can be explained by the fact that compounds with bulky substituents, such as methoxyl or acetyl groups (e.g., **21**, **28**, **37**, **39**, **43**, **47**, **49**, and **52**), have a greater occupation frequency than compounds with less bulky substituents (e.g., **19**, **23**, **30**, and **38**). In addition, these substituents are not able to perform hydrogen bonding interactions with Glu353 and Arg394 or they can sterically impair these interactions.

Finally, the GCOD (**1,6,−2**)(**np**) (Figure 4) is located in a 3D-box area that corresponds to the Ala350 residue. The negative coefficient of this GCOD (Figure 4) indicates that the occupation of this cell by non-polar atoms reduces the compound potency, probably due to steric factors. Although Figure 4 does not show clearly any atom of the ligands around this cell, some conformations and orientations adopted by the compounds during the MDS (data not shown) enable the carbon and hydrogen atoms of the piperidine ring to occupy this grid cell. It is important to notice that Figure 4 shows only one conformation, selected as the “bioactive” one, among the 2,000 conformers from the MDS of each compound, which leads to maximum potency according to Model **1B9**. In fact compounds with substituents at position 2' of the phenyl ring have low occupation frequency (e.g., **3**, **7**, **8**, and **16**), as well as compounds with substituents at positions 4 and 5 of the benzothiophene ring (e.g., **13**, **29**, **31**, **35**, **43**, **44**, **45**, **47**, and **49**). This fact indicates that such substituents try to maintain a favorable conformation of the basic side chain of the compounds to the antagonism towards ERα. This additional characteristic has not been revealed by the LIV-3D-QSAR Model developed by Cunha and co-workers using this series of compounds [18].

The absence of any descriptors around the phenyl ring, specifically related to the 4'-OH group, which is responsible for the hydrogen bond interaction with the backbone atoms of His524, corroborates what previous SAR studies already demonstrated [11,16], *i.e.*, the 6-OH group of the benzothiophenyl ring is more important for the biological activity than the 4'-OH group. However, the absence of any descriptors related to the 4'-position of the phenyl ring suggests some limitation of the model. Additionally, unlike observed in the LIV-3D-QSAR Model [18], the postulated “bioactive” conformation obtained in the 4D-QSAR Model **1B9** (Figure 4) is very similar to the one adopted by raloxifene in the X-ray co-crystal structure [3]. Comparing the compounds conformations from Model **1B9** with the raloxifene-ERα X-ray structure, the nitrogen atom of the piperidine ring is in a very close position to that one observed in the crystal, being the distance among them of 1.33Å for **1** (the most potent), and of 0.29Å for **54** (the least potent). These distances were calculated after RMS superposition of these compounds conformations over the X-ray structure of raloxifene bound in the LBD of ERα, according Alignment 1.

**Figure 5:**
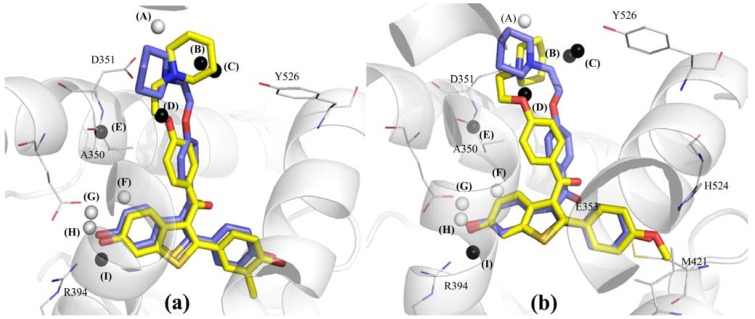
Graphical representation of compounds **9** (**a**) and **51** (**b**) according to the 4D-QSAR Model **1B9** (Alignment 1, grid cell size of 1.0 Å, and 9 terms). The postulated “bioactive” conformations of compounds **9** and **51** (stick models in yellow) are superposed (according Alignment 1) to the X-ray structure of raloxifene (stick models in blue) bound in the LBD of ERα (secondary structure and line models of selected amino acid residues in gray). GCODs of Model **1B9** are: (A) = (**1,13,1**)(**any**); (B) = (**2,11,4**)(**any**); (C) = (**0,11,5**)(**any**); (D) = (**−1,8,2**)(**any**); (E) = (**1,6,−2**)(np); (F) = (**1,2,0**)(**any**); (G) = (**0,1,−2**)(**any**); (H) = (**0,0,−2**)(**any**); and (I) = (**0,−2,−1**)(**any**). The white and black spheres represent the GCODs which occupation contributes to increase (GCODs A, F–H) or to decrease (GCODs B–E, and I) the potency of the compounds.

The SDres value for the training set was 0.38, which indicated compound 9 as an outlier (Figure 5 and Table 3). The only structural difference between this compound and raloxifene (1) is an additional substituent, 3'-Cl, at the phenyl ring of 9. According to Model 1B9, the predicted potency for compound 9 is lower than the experimental one, probably due to some limitation of the model, that does not show descriptors around the phenyl ring (as stated previously for the 4' position of the phenyl ring), or because of the presence of only few compounds with 3'-substituents, leading the model to underestimate the potency. Another possible explanation for the outlier behavior of compound 9 could be lipophilicity, which was not considered as a descriptor in this 4D-QSAR model. The calculated LogP value (cLogP) for the isomers containing one hydroxyl group in any position of the phenyl and benzothiophenyl rings is identical, e.g., the cLogP value for compounds **1**, **16**, **31**, **35**, and **41** is 5.96 [26]. However, the insertion of a chlorine atom in the phenyl ring increases the lipophilicity, since the cLogP value of compound **9** is 6.63.

Wang and co-workers [24] results indicated that LogP may not be essential to binding affinity on estrogen receptor α. However, the compounds have to be able to penetrate through cellular membranes. A 2D-QSAR [26] was performed to the same data set of this study, including LogP as a potential descriptor into the analysis. The authors concluded that the benzothiophene moiety is lipophilic enough to pass through this stage, which corroborate this hypothesis, since there is a significant positive correlation between potency and lipophilicity [25,26].

**Table 3:** Experimental (pIC_50Exp_) and calculated (pIC_50Calc_) potencies and residuals values (pIC_50Calc_—pIC_50Exp_) of Models **1B9** and **2B9** of 4D-QSAR.

# ^a^	pIC_50Exp_	Model 1B9		Model 2B9	
		pIC_50Calc_	Residue	pIC_50Calc_	Residue
**1**	9.70	9.84	0.14	9.27	−0.43
**2**	9.52	9.09	−0.43	8.11	−1.41 *
**3**	9.15	9.30	0.15	9.48	0.33
**4**	9.10	8.77	−0.33	9.04	−0.06
**5**	9.00	9.16	0.16	8.97	−0.03
**6**	9.00	8.49	−0.51	8.81	−0.19
**7**	8.70	8.57	−0.13	8.89	0.19
**8**	8.70	8.24	−0.46	8.32	−0.38
**9**	8.64	7.83	−0.81 *	8.83	0.19
**10**	8.64	8.45	−0.19	8.97	0.33
**11**	8.60	8.01	−0.59	8.32	−0.28
**12**	8.60	8.08	−0.52	8.59	−0.01
**13**	8.52	7.86	−0.66	8.47	−0.05
**14**	8.30	8.38	0.08	7.88	−0.42
**15**	8.15	8.09	−0.06	7.97	−0.18
**16**	8.00	8.39	0.39	7.86	−0.14
**17**	8.00	8.25	0.25	7.45	−0.55
**18**	7.70	7.85	0.15	7.76	0.06
**19**	7.70	7.41	−0.29	6.92	−0.78
**20**	7.52	7.81	0.29	7.58	0.06
**21**	7.52	7.62	0.10	7.41	−0.11
**22**	7.49	7.73	0.24	7.14	−0.35
**23**	7.46	7.10	−0.36	6.87	−0.59
**24**	7.40	7.46	0.06	7.86	0.46
**25**	7.30	7.89	0.59	7.28	−0.02
**26**	7.30	7.68	0.38	7.11	−0.19
**27**	7.30	6.93	−0.37	7.19	−0.11
**28**	7.22	6.76	−0.46	7.01	−0.21
**29**	7.00	7.08	0.08	6.45	−0.55
**30**	7.00	6.73	−0.27	6.59	−0.41
**31**	7.00	7.31	0.31	7.46	0.46
**32**	7.00	7.64	0.64	7.47	0.47
**33**	7.00	7.71	0.71	7.60	0.60
**34**	7.00	6.37	−0.63	6.86	−0.14
**35**	6.72	6.08	−0.64	6.27	−0.45
**36**	6.70	6.84	0.14	6.33	−0.37
**37**	6.60	6.53	−0.07	7.01	0.41
**38**	6.52	6.39	−0.13	6.91	0.39
**39**	6.52	6.02	−0.50	6.40	−0.12
**40**	6.52	6.63	0.11	6.32	−0.20
**41**	6.52	6.97	0.45	6.57	0.05
**42**	6.49	6.77	0.28	6.58	0.09
**43**	6.46	6.09	−0.37	6.39	−0.07
**44**	6.46	6.56	0.10	6.76	0.30
**45**	6.40	7.01	0.61	6.26	−0.14
**46**	6.30	6.15	−0.15	6.07	−0.23
**47**	6.30	6.18	−0.12	6.45	0.15
**48**	6.30	6.65	0.35	6.52	0.22
**49**	6.30	6.27	−0.03	7.44	1.14 *
**50**	6.22	6.43	0.21	6.40	0.18
**51**	6.00	7.12	1.12*	7.14	1.14 *
**52**	6.00	6.46	0.46	7.05	1.05 *
**53**	6.00	6.17	0.17	6.28	0.28
**54**	6.00	6.39	0.39	6.61	0.61

^a^ Underlined compounds’ numbers are those used in the test set. * Outlier compound (*i.e*., residuals higher than twice the standard deviation of the residual of fit).

As described in the previous section, the test data set were used to accomplish a “real” prediction using the 4D-QSAR Model **1B9**. The value of SD_res_ found for the test data set was 0.38, indicating compound **51** as an outlier (Table 3). The only structural difference between this compound and raloxifene (1) is the substitution of the 4'-OH of **1** by the 4'-OMe in **51**. The potency of compound **51** was overestimated by Model **1B9**. Again, this fact can be due to a limitation of the model, which does not present descriptors around the phenyl ring. Thus, it would not distinguish some putative unfavorable interaction of the methoxyl group with the neighboring residues (Figure 5). Besides, classic QSAR studies demonstrated that there is a negative steric effect for 4'-substituents in the phenyl ring [26].

### 2.4. Best Models from Alignment 2

The best models (**2B7**, **2B8**, and **2B9**) from Alignment 2 were obtained by 1.0 Å grid cells (Table 4). The cross-correlation matrix of the residues of the training set compounds according to models **2B7**, **2B8**, and **2B9** was calculated in order to eliminate models with the same type of information. High correlation among the residues of the three models (R > 0.5) is probably due to many identical cells, in terms of Cartesian coordinates and atom type occupation. The grid cells (**−1,−2,11**)(**any**) and (**0,2,2**)(**ar**) are present in all models, while the cells (**0,10,4**)(**any**) and (**0,5,−3**)(**any**) are present in models **2B7** and **2B8**. In other words, models **2B7**/**2B8** (R = 0.80) present four identical cells, while models **2B7**/**2B9** (R = 0.73) and **2B8**/**2B9** (R = 0.58) present two identical cells.

The cross-correlation matrix between experimental biological activity values and the grid cell occupancy of models **2B7**, **2B8**, and **2B9** (Table 5) indicates that the cells which present the highest correlation with the activity, are those found in Model **2B9**. These cells are (**0,10,**−**2**)(**any**) and (**0,2,2**)(**ar**), where the last one is present in the three models. Besides, those GCODs show poor correlation between themselves (R = 0.06).

**Table 4:** Best models of Alignment 2 obtained from 1.0 Å grid cells.

Model	Terms	Equation ^a^
**2B7**	7	**pIC_50_** = 7.70 + 2.47 (**−1,9,4**)(**any**) **−** 10.58 (**0,10,4**)(**any**) + 8.22 (**0,7,−2**)(**any**)
		− 5.27 (**−1,−2,11**)(**any**)**−**35.20 (**0,5,−3**)(**any**) + 21.70 (**0,2,2**)(**ar**)
		+ 0.84 (**0,−1,9**)(**ar**)
**2B8**	8	**pIC_50_** = 8.34 **−** 6.52 (**−3,9,4**)(**any**) **−** 9.04 (**0,10,4**)(**any**) **−** 7.64 (**0,2,−1**)(**any**) ****
		**−**5.08 (**−1,−2,11**)(**any**)**−**9.03 (**0,1,−2**)(**any**) + 5.37 (**−3,8,5**)(**any**)****
		**−**26.15 (**0,5,−3**)(**any**) + 23.89 (**0,2,2**)(**ar**)
**2B9**	9	**pIC_50_** = 6.66 **−** 30.48 (**−1,12,6**)(**any**) + 10.92 (**1,3,6**)(**hba**) + 16.70 (**0,11,3**)(**p−**) ****
		**−**15.21 (**0,12,−1**)(**any**) + 14.18 (**0,10,−2**)(**any**) + 0.57 (**0,0,−2**)(**any**)****
		**−**4.01 (**−1,−2,11**)(**any**) + 18.23 (**0,2,2**)(**ar**)**−**25.70 (**2,5,0**)(**any**)

^a^ The three numbers inside the first parenthesis defines the GCOD’s Cartesian coordinates (x,y,z), and the letters inside the second parenthesis defines the atom type occupancy (IPE).

**Table 5:** Cross-correlation matrix of the GCODs and the experimental pIC_50_ values of models **2B7**, **2B8**, and **2B9**.

	Potency		(−1,12,6) (any)				(1,3,6) (hba)		(0,11,3) (p−)			(0,12,−1) (any)			(−1,9,4) (any)			(0,10,−2) (any)			(−3,9,4) (any)			(0,10,4) (any)
**Potency**	1.00																							
**(−1,12,6)** **(any)**	−0.30		1.00																					
**(1,3,6)** **(hba)**	0.40		0.03				1.00																	
**(0,11,3)** **(p−)**	0.37		0.13				−0.19		1.00															
**(0,12,−1)** **(any)**	0.33		−0.11				−0.03		0.42			1.00												
**(−1,9,4)** **(any)**	−0.15		0.03				−0.15		−0.13			−0.26			1.00									
**(0,10,−2)** **(any)**	0.49		−0.06				0.28		0.29			0.73			−0.13			1.00						
**(−3,9,4)** **(any)**	−0.21		−0.27				−0.33		−0.28			−0.33			0.59			−0.27			1.00			
**(0,10,4)** **(any)**	−0.50		0.71				−0.29		−0.19			−0.28			0.31			−0.30			0.21			1.00
	**Potency**		**(−1,12,6)** **(any)**		**(1,3,6)** **(hba)**			**(0,11,3)** **(p−)**			**(0,12,−1)** **(any)**			**(−1,9,4)** **(any)**			**(0,10,−2)** **(any)**			**(−3,9,4)** **(any)**			**(0,10,4)** **(any)**	**(0,7,−2)** **(any)**
**(0,7,−2)** **(any)**	0.22		−0.14		0.26			0.14			0.22			−0.10			0.47			−0.24			−0.34	1.00
**(0,2,−1)** **(any)**	−0.16		0.12		0.07			−0.11			0.01			0.00			0.07			−0.09			0.08	−0.03
**(0,0,−2)** **(any)**	0.41		−0.07		0.24			0.16			0.13			−0.24			0.23			−0.22			−0.38	0.29
**(−1,−2,11)** **(any)**	−0.34		0.13		0.02			−0.21			−0.16			−0.08			−0.14			−0.04			0.16	−0.07
**(0,1,−2)** **(any)**	−0.28		0.46		−0.03			-0.05			0.06			−0.19			−0.02			−0.14			0.24	−0.14
**(−3,8,5)** **(any)**	−0.21		−0.29		−0.33			−0.26			−0.29			0.69			−0.15			0.92			0.16	−0.15
**(0,5,−3)** **(any)**	−0.22		−0.12		0.06			0.01			−0.25			−0.05			−0.18			−0.11			−0.21	0.59
**(0,2,2)** **(ar)**	0.45		0.10		0.44			0.06			0.03			−0.16			0.06			−0.16			−0.04	0.09
**(2,5,0)** **(any)**	−0.30		−0.10		−0.01			−0.05			−0.30			0.06			−0.26			0.01			−0.09	0.47
**(0,−1,9)** **(ar)**	0.15		−0.09		0.02			−0.02			−0.25			−0.18			−0.24			−0.05			−0.01	0.08
		**(0,2,−1)** **(any)**		**(0,0,−2)** **(any)**		**(−1,−2,11)** **(any)**				**(0,1,−2)** **(any)**			**(−3,8,5)** **(any)**			**(0,5,−3)** **(any)**			**(0,2,2)** **(ar)**			**(2,5,0)** **(any)**		**(0,−1,9)** **(ar)**
**(0,7,−2)** **(any)**																								
**(0,2,−1)** **(any)**		1.00																						
**(0,0,−2)** **(any)**		−0.04		1.00																				
**(−1,−2,11)** **(any)**		−0.08		−0.30		1.00																		
**(0,1,−2)** **(any)**		−0.09		−0.16		0.22				1.00														
**(−3,8,5)** **(any)**		−0.05		−0.26		−0.01				−0.15			1.00											
**(0,5,−3)** **(any)**		−0.05		0.16		0.00				−0.12			−0.06			1.00								
**(0,2,2)** **(ar)**		0.04		0.12		0.30				0.11			−0.20			−0.01			1.00					
**(2,5,0)** **(any)**		−0.01		0.08		0.00				−0.14			0.07			0.94			−0.07			1.00		
**(0,−1,9)** **(ar)**		−0.01		0.08		0.20				−0.11			−0.05			0.22			0.44			0.22		1.00

Model **2B7** presents a higher correlation with the other models, especially with model **2B8** (R = 0.80). In a first analysis, Model **2B7** incorporates the other models quantitatively, because it presents more identical cells as compared to the other models. However, models **2B8** and **2B9** are less correlated between themselves (R = 0.58) and, at the same time, highly correlated to model **2B7**. Therefore, an additional criteria was taken into account in order to select the representative Model of Alignment 2, *i.e.*, the SD_res_ value and the number of outliers. We observed that model **2B9** presents a lower value of SD_res_, in spite of presenting the same number of outliers considering the training set compounds. Besides, this model also possesses the highest values of R^2^_adj_ and Q^2^_adj_, being selected as the most representative model of Alignment 2.

### 2.5. Graphical Analysis of the Representative Model of Alignment 2

**Figure 6:**
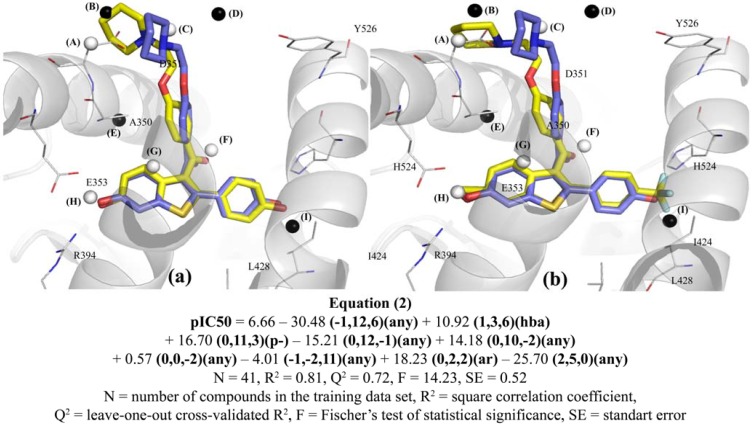
Graphical representation of compounds **1** (**a**) and **54** (**b**) according to the 4D-QSAR Model **2B9** (Alignment 2, grid cell size of 1.0 Å, and 9 terms). The postulated “bioactive” conformations of compounds **1** and **54** (stick models in yellow) are superposed (according Alignment 2) to the X-ray structure of raloxifene (stick models in blue) bound in the LBD of ERα (secondary structure and line models of selected amino acid residues in gray). GCODs of Model **2B9** are (A) = (**0,10,−2**)(**any**); (B) = (**0,12,−1**)(**any**); (C) = (**0,11,3**)(**p−**); (D) = (**−1,12,6**)(**any**); (E) = (**2,5,0**)(**any**); (F) = (**1,3,6**)(**hba**); (G) = (**0,2,2**)(**ar**); (H) = (**0,0,−2**)(**any**); and (I) = (**−1,−2,11**)(**any**). The white and black spheres represent the GCODs which occupation contributes to increase (GCODs A, C, F, G, and H) or to decrease (GCODs B, D, E, and I) the potency of the compounds.

Model **2B9** (described in Equation 2) was selected as the most representative model of Alignment 2, as previous reported in the selection criteria outlined above. According to the cross-correlation matrix of grid cell occupancy of model **2B9** (Table 5), the descriptors are nearly orthogonal and contribute in a different way to the 4D-QSAR models, except for only two pairs of GCODs. The GCODs (**0,10,−2**)(**any**) and **(0,12,−1**)(**any**) shows high correlation (R = 0.73). Although the GCOD (**0,10,−2**)(**any**) occupation increases the compounds potency and the GCOD (**0,12,−1**)(**any**) occupation decreases, these cells are close in space (distance of 2.24 Å), what would justify the correlation between them. The GCOD (**0,10,−2**)(**any**) shows an ambiguity, because it is located in an area of the 3D grid cell close to Asp351 (Figure 6). Therefore, it is not expected that this GCOD occupation increases the compounds potency. It demonstrates that model **2B9** is unable to “predict” the presence of Asp351 and the attractive electrostatic interaction between this residue and the piperidine group of raloxifene, as can be noticed by visual inspection of the 3D structure of the raloxifene-ERα complex (PDB code 1ERR [3]). This data may be used to rationalize the underestimation of the potency of compound **1**, the most potent compound of the series under study.

The second pair of GCODs that shows medium correlation (R = 0.44) corresponds to GCODs (**0,2,2**)(**ar**) and **(1,3,6**)(**hba**). Unlike the previous case, those descriptors are distant in space (distance of 4.24Å) and both contribute to increase the potency. The GCOD (**0,2,2**)(**ar**) is located close to the carbon and hydrogen atoms at position 4 of the benzothiophene ring, while the GCOD (**1,3,6**)(**hba**) is located close to the oxygen atom of the carbonyl group. Those GCODs are related to the dihedral angle formed by the carbonyl and benzothiophenyl planes, indicating the importance of the coplanar orientation of the side chain, as described by Grese and coworkers [11]. The molecules that occupy these cells most frequently are those that present substituents at position 2' of the phenyl ring, e.g., compounds **3**, **7**, **8**, and **16**. Substituents at these positions generate steric repulsion with the oxygen atom of the carbonyl group, leading the side chain to adopt a non-coplanar orientation.

GCODs (**−1,12,6**)(**any**) and (**0,11,3**)(**p−**) are located close to the piperidine ring, corroborating the importance of the side chain orientation. The occupation of GCOD (**−1,12,6**) by any atom type decreases the potency of the compounds, displacing the nitrogen atom of the piperidine group from the favorable position, related to the hydrogen bonding with residue Asp351. The GCOD (**0,11,3**)(**p−**) occupation is also related to the nitrogen atom of the side chain, since substituents at position 4 of the benzothiophene ring or at position 3' of the phenyl group have a high occupation frequency at this cell, e.g., compounds **5**, **9**, **29**, **35**, and **44**. This indicates that these substituents are able to maintain a favorable conformation of the basic side chain of the compounds to the antagonism on ERα.

The GCOD (**2,5,0**)(**any**) (Figure 6), located close to the residue Ala350, has the same behavior of grid cell (**1,6,**−**2**)(**np**) from Model **1B9**.

GCOD (**0,0,**−**2**)(**any**), located close to Glu353, indicates the importance of hydrogen bonding of this residue with the substituent at position 6 of the benzothiophene ring. This grid cell has a low frequency of occupation when there are no substituents at this position (e.g., compounds **23**, **30** and **38**) or the substituents are unable to perform this type of interaction (e.g., compound **19**).

The GCOD (−**1,**−**2,11**)(**any**), located close to position 4' of the phenyl group, is related to the nonpolar interactions around this ring. As this area is wrapped up by hydrophobic residues (e.g., Ile424, Gly521, and Leu525), compounds with bulky substituents at this position (e.g., compounds **22**, **33**, **36**, **39**, **43** and **51**) show steric hindrance and, consequently, lower potency.

**Figure 7:**
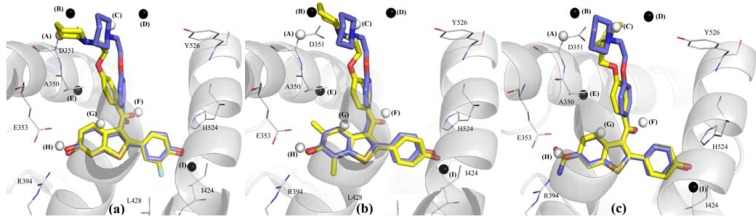
Graphical representation of compounds **2** (**a**), **49** (**b**) and **52** (**c**) according to the 4D-QSAR Model **2B9** (Alignment 2, grid cell size of 1.0 Å, and 9 terms). The postulated “bioactive” conformations of compounds **2**, **49** and **52** (stick models in yellow) are superposed (according Alignment 2) to the X-ray structure of raloxifene (stick models in blue) bound in the LBD of ERα (secondary structure and line models of selected amino acid residues in gray). GCODs of Model **2B9** are (A) = (**0,10,−2**)(**any**); (B) = (**0,12,−1**)(**any**); (C) = (**0,13,3**)(**p−**); (D) = (**−1,12,6**)(**any**); (E) = (**2,5,0**)(**any**); (F) = (**1,3,6**)(**hba**); (G) = (**0,2,2**)(**ar**); (H) = (**0,0,−2**)(**any**); and (I) = (**−1,−2,11**)(**any**). The white and black spheres represent the GCODs which occupation contributes to increase (GCODs A, C, F, G, and H) or to decrease (GCODs B, D, E, and I) the potency of the compounds.

Three compounds of the training set were identified as outliers: **2**, **49** and **52** (Table 3). The predicted activity for compound **2** is lower than the experimental one. The structural difference between this compound and raloxifene (**1**) is an additional substituent, 3'-F, at the phenyl ring of **2**. Like Model **1B9**, this fact can be due to the existence of few compounds with substituents at position 3' of the phenyl ring. Therefore, the model does not reveal the importance of the substitution pattern at this position. Compounds **49** and **52** show higher predicted potencies than the experimental ones. The chemical difference between raloxifene (**1**) and **49** is the presence of two additional substituents, 5,7-Me, at the phenyl ring. Model **2B9** does not show any descriptors around those substituents, which turns the model unable to recognize how they may influence the conformation of the side chain, leading to an unfavorable orientation for potency (Figure 7). Compound **52** has an amide substituent at position 6 of the benzothiophene ring that has a high frequency of occupation of GCOD (**0,0,**−**2**)(**any**). However, this cell presents a low coefficient (Equation 2), which tends to have a small contribution to Model **2B9**.

Compound **51**, from test data set, was identified as an outlier and its potency was overestimated by Model **2B9** (Table 3). The same behavior was observed in Model **1B9**. In a different fashion as observed with the representative model of alignment 1, Model **2B9** has a close descriptor at position 4' of the phenyl ring, *i.e.*, GCOD **(−1,−2,11)(any)**. This fact supports 2D-QSAR data about the negative steric effect of substituents at this position [26]. This GCOD is also occupied by compound **51**, but its contribution to the model is not significant, due to its small coefficient value when compared to other GCODs.

**Figure 8:**
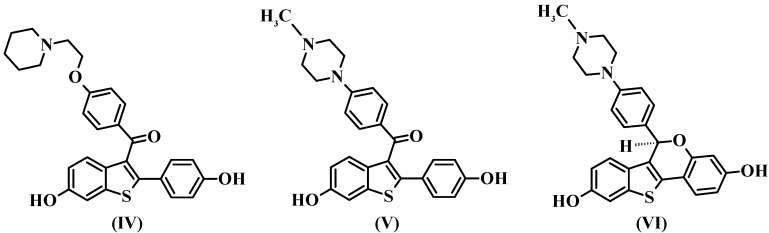
Structures of the raloxifene **IV** (pIC_50Exp_ = 9.70 M) and the proposed compounds **V** and **VI** (and calculated potencies based on the 4D-QSAR Model **1B9**).

Although alignment 2 has considered atoms belonging to more rigid regions of the molecules and shows descriptors better distributed in space, alignment 1 has superior statistical indices and is more consistent with the considered raloxifene mechanism of action. Thus, based in the results of the 4D-QSAR Model **1B9** and in a previous LIV-3D-QSAR model from our group [18], the synthesis of new SERMs candidates has been suggested (Figure 8).

### 2.6. New Compounds Based on 4D-QSAR Analysis

In medicinal chemistry, the optimization of lead compounds proceeds along two main methods [27]. The first one is based on chemical modifications of the molecular structure (changing the chemical properties of the molecule), which have been exhaustively explored by Grese and co-workers [16] in this series of raloxifene analogs. The second one is the application of conformational constraints that change the molecular flexibility. Therefore, based on the results obtained by Model **1B9** of the current 4D-QSAR analysis and in the previous LIV-3D-QSAR study [18], we suggest two modifications on the raloxifene structure in order to reduce side chain flexibility. (i) The piperidinyl-ethoxy moiety was replaced by a piperazine group (proposed compounds **V** and **VI**), which is able to maintain the electrostatic and the hydrogen bonding interactions with Asp351, since this group has a basic nitrogen atom at the same position in relation to raloxifene. (ii) The carbonyl “hinge” and the phenolic group were replaced by a naphthyl group (proposed compound **VI**).

According to the 4D-QSAR Model **1B9**, compound **V** showed the poorest calculated potency (pIC_50_ = 6.60), while compound **VI** has shown the highest calculated potency (pIC_50_ = 10.48) (Figure 8). Replacing the basic side chain and maintaining the carbonyl “hinge” (**V**) may lead to side chain orientations, which are not favorable to biological activity, since the basic side chain follows the carbonyl “hinge” orientation. Therefore, GCODs that contributes negatively to the potency have a high frequency of occupation.

A SAR study from 1997 [11], indicated that nearly orthogonal orientations of the basic side chain in raloxifene might be responsible for its unique biological activity profile. However, it is interesting to note that the most potent proposed compound (**VI**) shows a coplanar orientation of the basic side chain. This result may be due to its similarity to the bioactive conformation of raloxifene, since the RMS deviation value (0.28 Å) between **VI** and the raloxifene X-ray crystallographic structure is low. Moreover, this result shows that the simultaneously replacement of both the basic side chain and the carbonyl “hinge” (**VI**) leads to a rigid conformation of the lateral chain, which does not allow unfavorable orientations. Therefore, GCODs that contributes positively to the potency have a high frequency of occupation. It is interesting to note that, benzothiophene [28] and tetrahydrolsoquinoline [29] derivatives containing a constrained piperazine side chain were reported as high-aﬃnity ligands of the ERα, being potent agonists in bone tissue.

## 3. Experimental

### 3.1. Computational Methods

#### 3.1.1. Biological Data

The 4D-QSAR analysis [20] was applied to a series of 54 raloxifene analogs [16], using a training data set of 41 compounds (**1**–**7**, **9**–**13**, **15**, **16**, **19**, **21**–**25**, **27**–**30**, **32**, **34**–**39**, **42**–**45**, **47**, **49**, **50** and **52**–**54**), randomly selected from the original 54 compounds, which were used for model construction and internal validation (cross-validation). The model was also externally validated using a test data set of 13 compounds (**8**, **14**, **17**, **18**, **20**, **26**, **31**, **33**, **40**, **41**, **46**, **48** and **51**). Table 3 reports the 54 compounds’ structures and the related potencies, defined as IC_50_ (nM), where C is the compound effective inhibitory concentration required to achieve 50% (IC_50_) inhibition of MCF-7 cell proliferation [16]. The IC_50_ values (nM) were transformed into pIC_50_ (M) values (−LogIC_50_).

#### 3.1.2. Structures Building

The three-dimensional (3D) models of the 54 compounds (Table 6) were based on the structure of compound **1** (raloxifene) co-crystallized with ER, retrieved from the Protein Data Bank (PDB code: 1ERR) [15], corresponding to the putative “bioactive” conformation. The 3D model for each compound was built with the nitrogen atom of the piperidine group protonated, using HyperChem 7.0 software [30]. Each structure, including raloxifene (**1**), was geometry-optimized in vacuum, without any restriction, using the MM+ molecular mechanics force field (HyperChem) [31], and subsequently using the semi-empirical AM1 Hamiltonian (HyperChem) [32], in order to assign the partial atomic charges.

**Table 6:** Chemical structures and pIC_50_ (M) values (−LogIC_50_) of a series of 54 raloxifene analogs [12]. Underlined compounds’ numbers are those used in the test set. 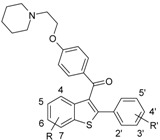

#	R	R'	pIC_50_		#	R	R'	pIC_50_
**1**	6-OH	4'-OH	9.70		**28**	6-COMe	4'-OH	7.22
**2**	6-OH	3'-F, 4'-OH	9.52		**29**	4,7-di(Me), 6-OH	4'-OH	7.00
**3**	6-OH	2'-Me	9.15		**30**	H	4'-OMe	7.00
**4**	6-OH	4'-C≡CH	9.10		**31**	5-OH	4'-OH	7.00
**5**	6-OH	3'-Me, 4'-OH	9.00		**32**	6-OH	4'-Ph	7.00
**6**	6-OH	4'-Cl	9.00		**33**	6-OH	4'-CH_2_SEt	7.00
**7**	6-OH	2'-Me, 4'-OH	8.70		**34**	6-OH	3',5'-di(Me), 4'-OH	7.00
**8**	6-OH	2'-OMe, 4'-OH	8.70		**35**	4-OH	4'-OH	6.72
**9**	6-OH	3'-Cl, 4'-OH	8.64		**36**	6-OH	4'-CONH_2_	6.70
**10**	6-OH	4'-F	8.64		**37**	6-OMe	4'-OH	6.60
**11**	6-OH	H	8.60		**38**	H	H	6.52
**12**	6-OH	3'-F	8.60		**39**	6-OMe	4'-OMe	6.52
**13**	5-F, 6-OH	4'-OH	8.52		**40**	6-Me	4'-OH	6.52
**14**	6-OH	4'-Et	8.30		**41**	7-OH	4'-OH	6.52
**15**	6-OH	4'-CH=CH_2_	8.15		**42**	6-OH	4'-CO_2_H	6.49
**16**	6-OH	2'-OH	8.00		**43**	5,6,7-tri(OMe)	4'-OMe	6.49
**17**	6-OH	4'- *n*-Bu	8.00		**44**	4,6-di(OH)	4'-OH	6.46
**18**	6-OH	4'-CONMe_2_	7.70		**45**	5,6-di(OH)	4'-OH	6.40
**19**	6-C≡CH	4'-OH	7.70		**46**	6-OH	4'-NO_2_	6.30
**20**	6-OH	4'- *i*-Pr	7.52		**47**	4,5-benzo, 6-OH	4'-OH	6.30
**21**	6-CO_2_Me	4'-OH	7.52		**48**	6-OMe	3',4'-OCH_2_O-	6.30
**22**	6-OH	4'-COMe	7.49		**49**	5,7-di(Me), 6-OH	4'-OH	6.30
**23**	H	4'-OH	7.46		**50**	6-OMe	4'-CH_2_OH	6.22
**24**	6-OH	4'-CONHMe	7.40		**51**	6-OH	4'-OMe	6.00
**25**	6-OH	4'-Me	7.30		**52**	6-CONH_2_	4'-OH	6.00
**26**	6-OH	4'-CO_2_Me	7.30		**53**	6-Cl	4'-OH	6.00
**27**	6-OH	4'-CO_2_Et	7.30		**54**	6-OH	4'-CF_3_	6.00

#### 3.1.3. Conformational Sampling

Molecular dynamics simulation (MDS) was carried out using the MOLSIM package in the 4D-QSAR program [33], starting from the AM1 structures, in order to construct the conformational ensemble profile (CEP) of each ligand. The temperature for MDS was set at 310 K with a simulation sampling time of 100 ps and time step of 0.001 ps. The atomic coordinates of each conformation during the MDS were recorded every 50 steps to generate 2,000 conformations of each analog.

#### 3.1.4. Interaction Pharmacophore Elements (IPEs) Definition

The 4D-QSAR methodology defines seven types of interaction pharmacophore elements (IPEs), which correspond to atom types that may occupy the grid cells and interact into the binding site: (i) any type (any); (ii) nonpolar (np); (iii) polar-positive charge (p+); (iv) polar-negative charge (p−); (v) hydrogen bond acceptor (hba); (vi) hydrogen bond donor (hbd) and (vii) aromatic systems (ar).

#### 3.1.5. Grid Cell Sizes Definition

In this study, grid cell sizes of 2.0 and 1.0 Å were explored. The 2.0 Å grid cell size corresponds to the integral number closest to twice the hydrogen atom van der Waals radii (1.2 Å) and, thus, is large enough to encompass a hydrogen atom [34]. The 1.0 Å grid cell size was used with the intention to refine the 2.0 Å grid cell 4D-QSAR models.

#### 3.1.6. Alignment Definition

Three alignments were used to define the lattice overlay of the CEP of each compound. In alignments 1 and 2, the atoms were selected based on previous SAR studies from the literature [3,11,16], while in alignment 3, we used non-pharmacophoric atoms to observe if the 4D-QSAR program is able to select the most important IPEs. The atom numbers and corresponding sequences for each alignment are listed in Table 7, using the raloxifene structure (compound **1**) as a template.

**Table 7:** Atom numbering of the tested alignments used in 4D-QSAR models’ construction.

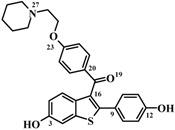	**Alignment**	**1st Atom ^a^**	**2nd Atom ^a^**	**3rd Atom ^a^**
	**1**	C3	C12	N27
	**2**	C3	C9	C23
	**3**	C16	O19	C20

^a^ The atom numbers of raloxifene (compound **1**) were automatically obtained from HyperChem numbering [17] and not by IUPAC rules.

#### 3.1.7. Independent Variable Generation

The CEP for each compound was overlaid onto a cubic lattice of a selected grid cell size (2.0 and 1.0 Å), according to each alignment. The grid cell occupancy profile for each IPE was computed and used as the 4D-QSAR descriptors, which are named Grid Cell Occupancy Descriptors (GCODs). Thus, the normalized grid cell absolute occupancy [20,23], defined as the number of times a cell was occupied by an atom type over the MDS, divided by the size of the CEP (2,000 conformations), was used to define the GCODs.

#### 3.1.8. Data Reduction

The 4D-QSAR analysis, like other 3D-QSAR methods, generates an great number of QSAR variables (GCODs) because of the large number of grid cells and the number of IPEs [20,34]. Thus, three serial levels of data reduction were considered, in order to eliminate spurious variables [34]. The first level eliminated GCODs that have an individual correlation coefficient, R, with the activity values less than 0.1; the second level eliminated GCODs whose variance (self-variance) over a set of analogs was less than a prechosen fraction; and the third level eliminated GCODs with a prechosen number of empty grid cells (Table 8).

**Table 8:** Data reduction before the 4D-QSAR analyses according to the alignment and grid cell size.

Alignment	Grid Cell Size (Å)	Self-variance	Empty Grid Cells
**1**	2.0	<0.00800	>37
**1**	1.0	<0.00400	>37
**2**	2.0	<0.00500	>37
**2**	1.0	<0.00015	>36
**3**	2.0	<0.00500	>38
**3**	1.0	<0.00018	>36

#### 3.1.9. 4D-QSAR Model Construction

The 4D-QSAR models building and optimization process employed the Genetic Function Approximation (GFA) [35] coupled with Partial Least Squares (PLS) regression [36]. Improved models are constructed by performing crossover operations to recombine the descriptors of the better-scored models, according to the Friedman’s “lack-of-fit” (LOF) measure, which penalizes the Least Square Error (LSE) measure [34,35]. The number of crossover operations was set from 6,000 to 20,000. In addition, mutation probability was set at 100% and a smoothing factor (the variable that controls the number of independent variables in the models) ranged from 0.5 to 3.0.

#### 3.1.10. Internal and External 4D-QSAR Model Validation

The best-scored GA-PLS equations were submitted to internal validation by “leave-one-out” cross-validation (LOO-cv) technique in the 4D-QSAR program. LOO-cv correlation coefficient (Q^2^), squared correlation coefficient (R^2^), standard error (SE), and Fischer’s test (F) were used as parameters to select the best models. The test data set (13 compounds) was used to test the best 4D-QSAR models for their ability to predict biological activity values of compounds not included in the training data set.

#### 3.1.11. Bioactive Conformation Selection

The final step in the 4D-QSAR methodology is to hypothesize the *bioactive conformation* of each compound in the training set. The lowest-energy conformer state (up to 10.0 kcal/mol from the minimum energy conformation), which predicted the maximum potency, using the optimum 4D-QSAR model, was defined as the “bioactive” conformation.

## 4. Conclusions

A series of 54 raloxifene analogs, evaluated as estrogen receptor-α ligands, was selected from the literature for a 4D-QSAR study, applying three tentative alignments and grid cells of 2.0 and 1.0 Å. The best models were obtained from alignments 1 and 2, using grid cell size of 1.0 Å, from a training set of 41 compounds. In addition, a test set of 13 compounds were used in the external validation process. The best models were also validated based on the biological system and mechanism of action of the compounds under study.

The models generated by 1.0 Å grid cell are more predictive, since they showed higher Q^2^_adj_ values than the best models from 2.0 Å grid cell, irrespective to the alignment. The models from both alignments 1 and 2 were also consistent with the ER modulators action mechanism. A representative model was selected for each one of alignments 1 (Model **1B9**) and 2 (Model **2B9**), revealing the degree in which the lateral chain flexibility of the raloxifene analogs influences the potency.

Although there are any descriptors associated to the 4'-position of the phenyl ring, it is the most coherent with the X-ray crystallography data. The model **2B9** was incapable to preview the presence of Asp351, which has an important contribution to binding activity of raloxifeno derivatives on estrogen receptor α. Both models do not consider cLogP as a descriptor and this limitation can explain the outlier compounds behavior.

In order to evaluate the influence of the reduction of the side chain flexibility on the potency and based on the results from the 4D-QSAR analysis, we proposed two new raloxifene analogs based on the model **1B9**. The results indicated that the highest degree of rigidity imposed to the lateral side chain increases the calculated potency, since it does not allow unfavorable orientations, maintaining most of the time the favorable electrostatic and hydrogen bond interactions with Asp351. Therefore, the drastic reduction of the side chain flexibility and, consequently, the generation of more favorable conformations of compounds to achieve better interactions with the receptor may be a successful strategy.
